# Supplementary material for: Nothing but hot air?—On the molecular ballistic analysis of backspatter generated by and the hazard potential of blank guns
Source: Int J Legal Med. 2021 Mar 8;135(5):2061–71. doi: 10.1007/s00414-021-02541-y (PMC8354942; doi:10.1007/s00414-021-02541-y)
Supplement: Supplementary file 1 — (PPTX 24719 kb) [file 414_2021_2541_MOESM1_ESM.pptx]

## Slide 1
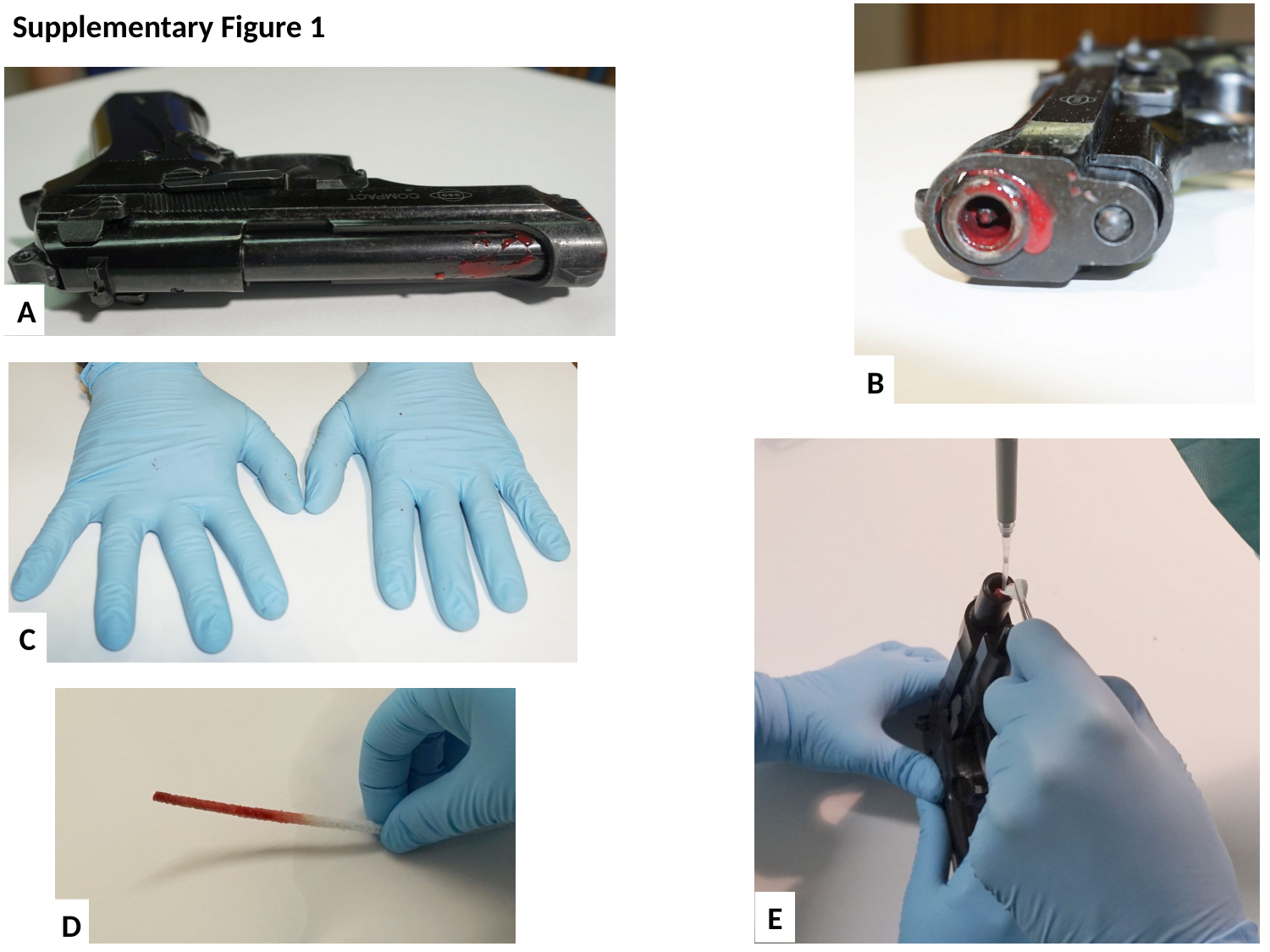

Supplementary Figure 1
A
B
C
E
D

## Slide 2
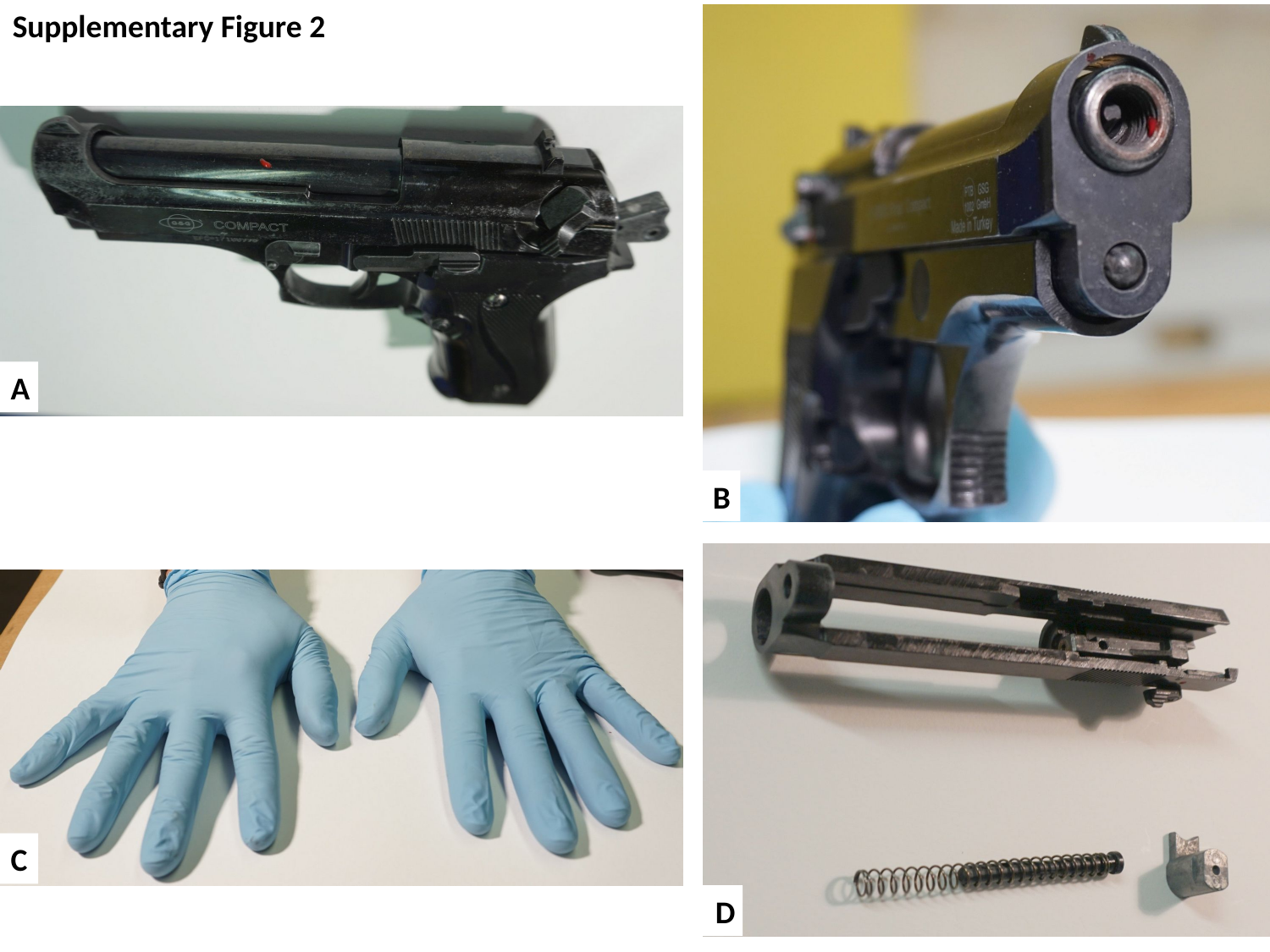

Supplementary Figure 2
A
B
C
D

## Slide 3
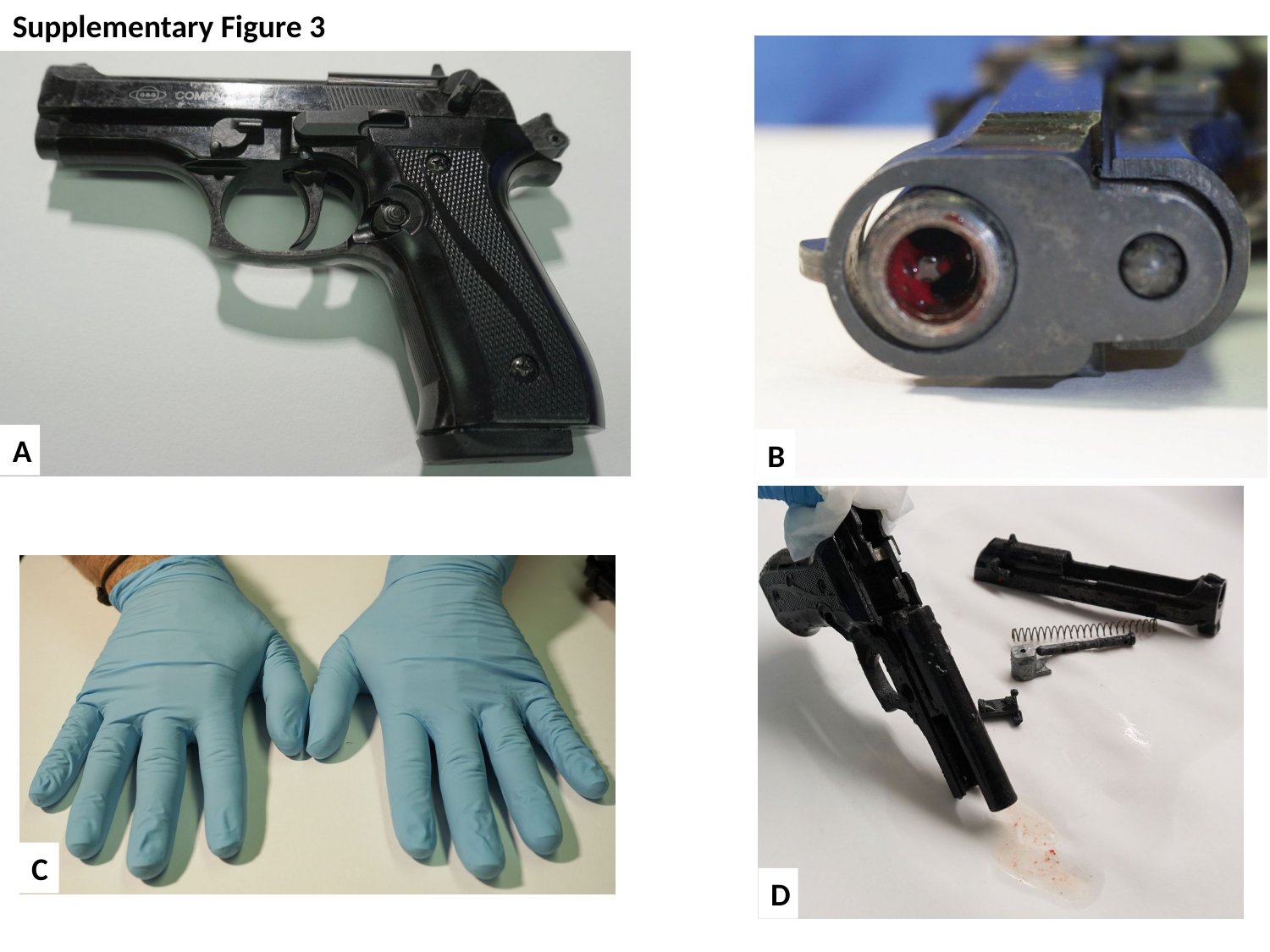

Supplementary Figure 3
A
B
C
D

## Slide 4
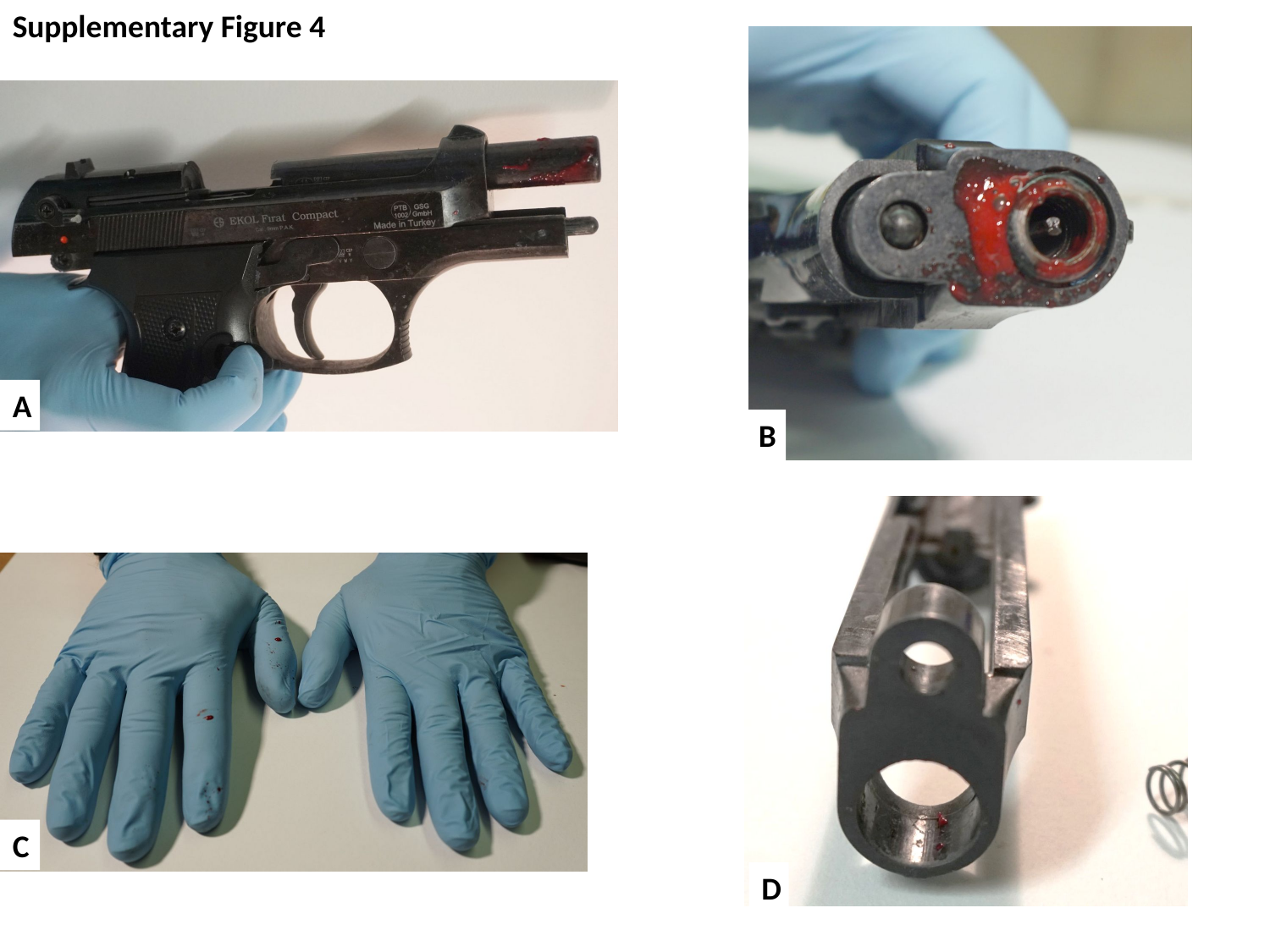

Supplementary Figure 4
A
B
C
D

## Slide 5
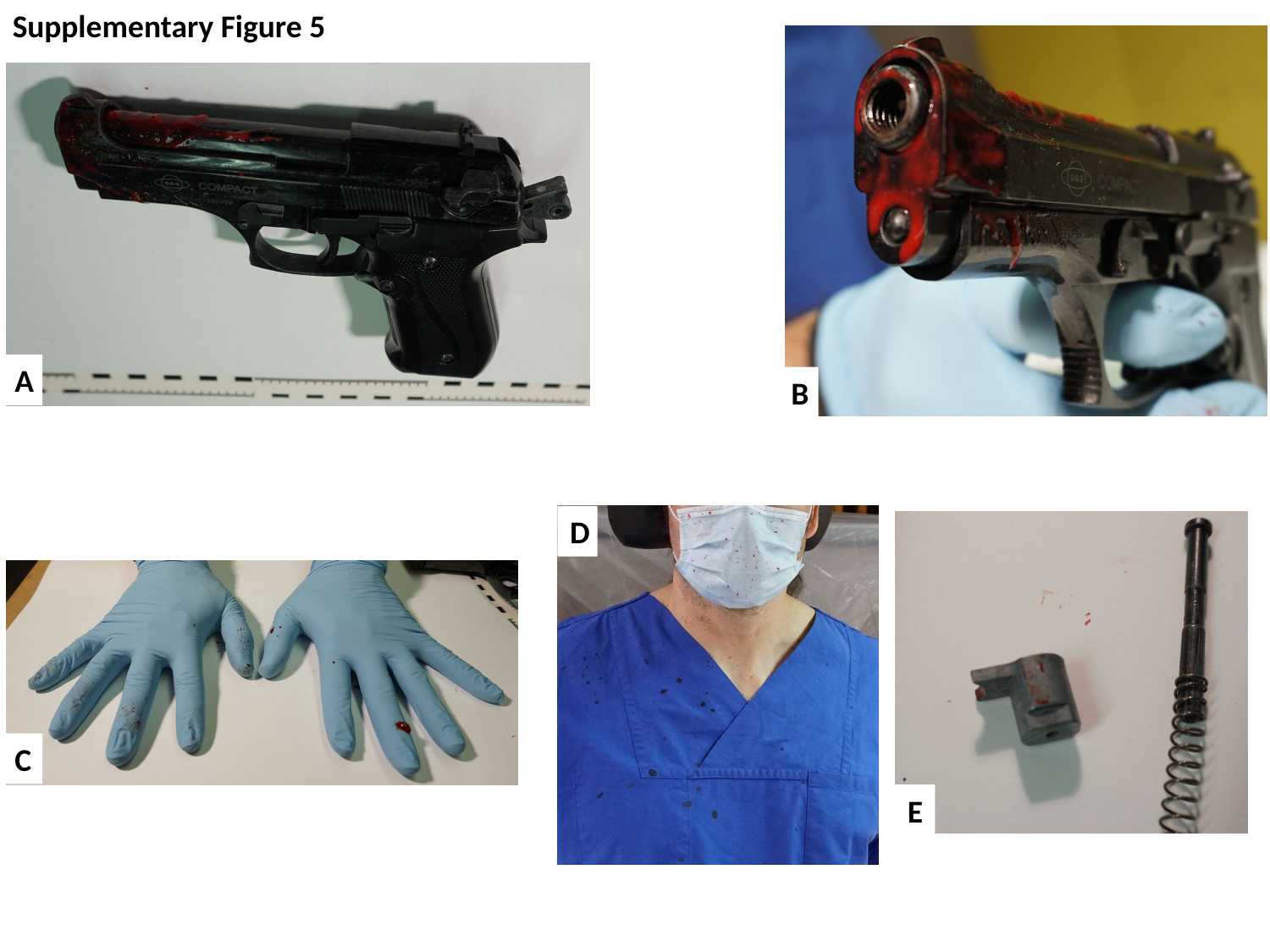

Supplementary Figure 5
A
B
D
C
E

## Slide 6
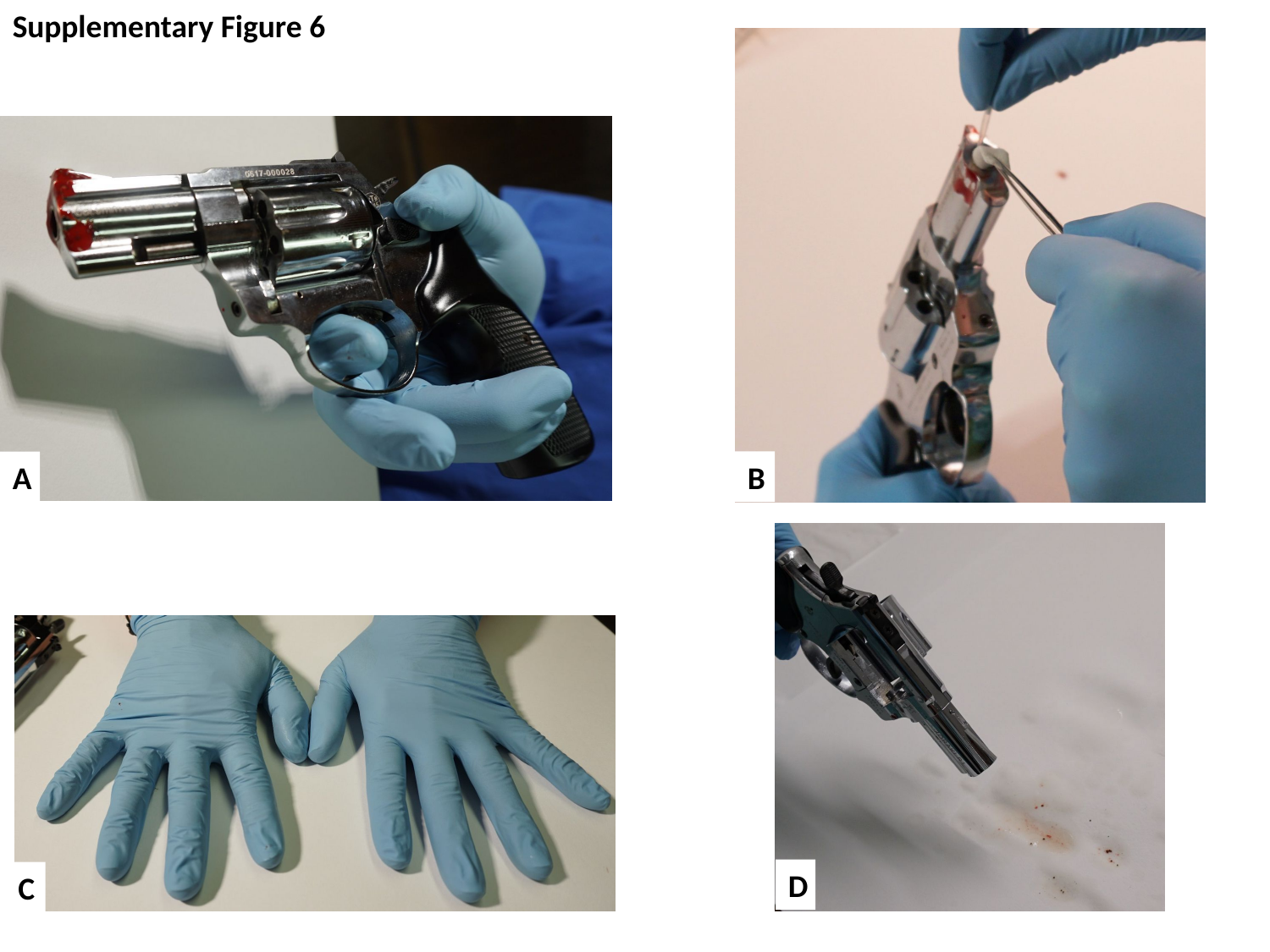

Supplementary Figure 6
B
A
D
C

## Slide 7
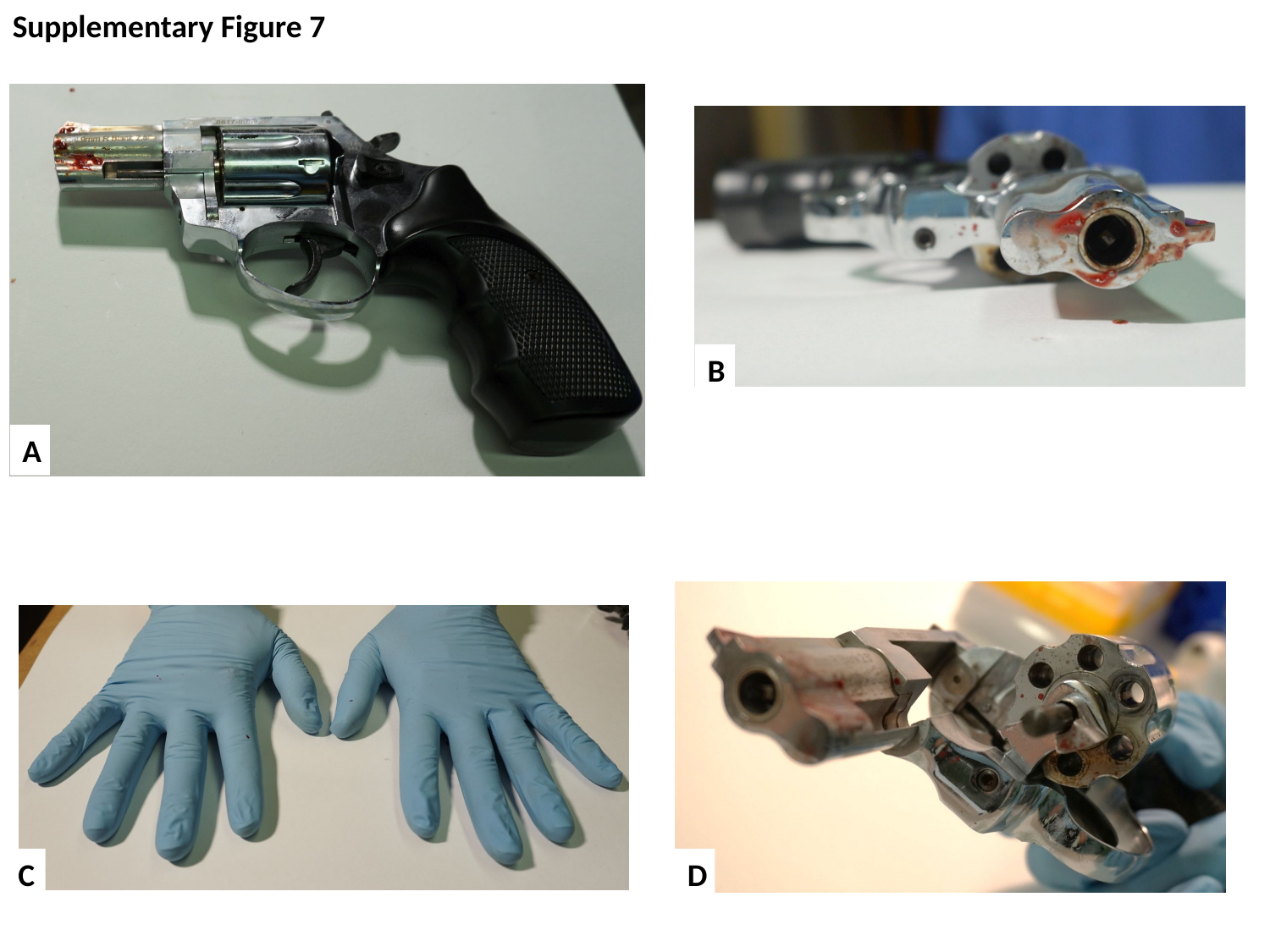

Supplementary Figure 7
B
A
C
D

## Slide 8
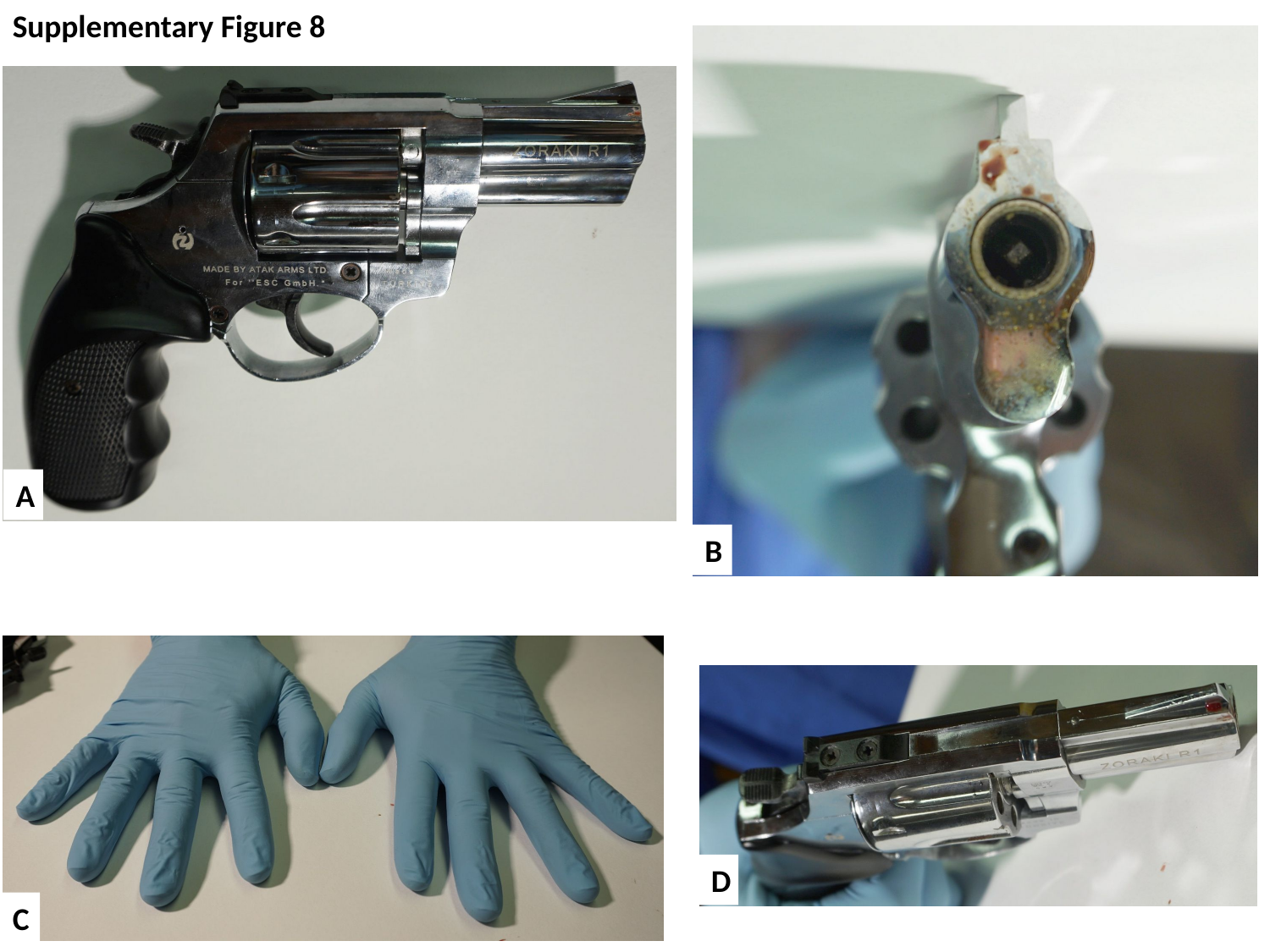

Supplementary Figure 8
A
B
D
C

## Slide 9
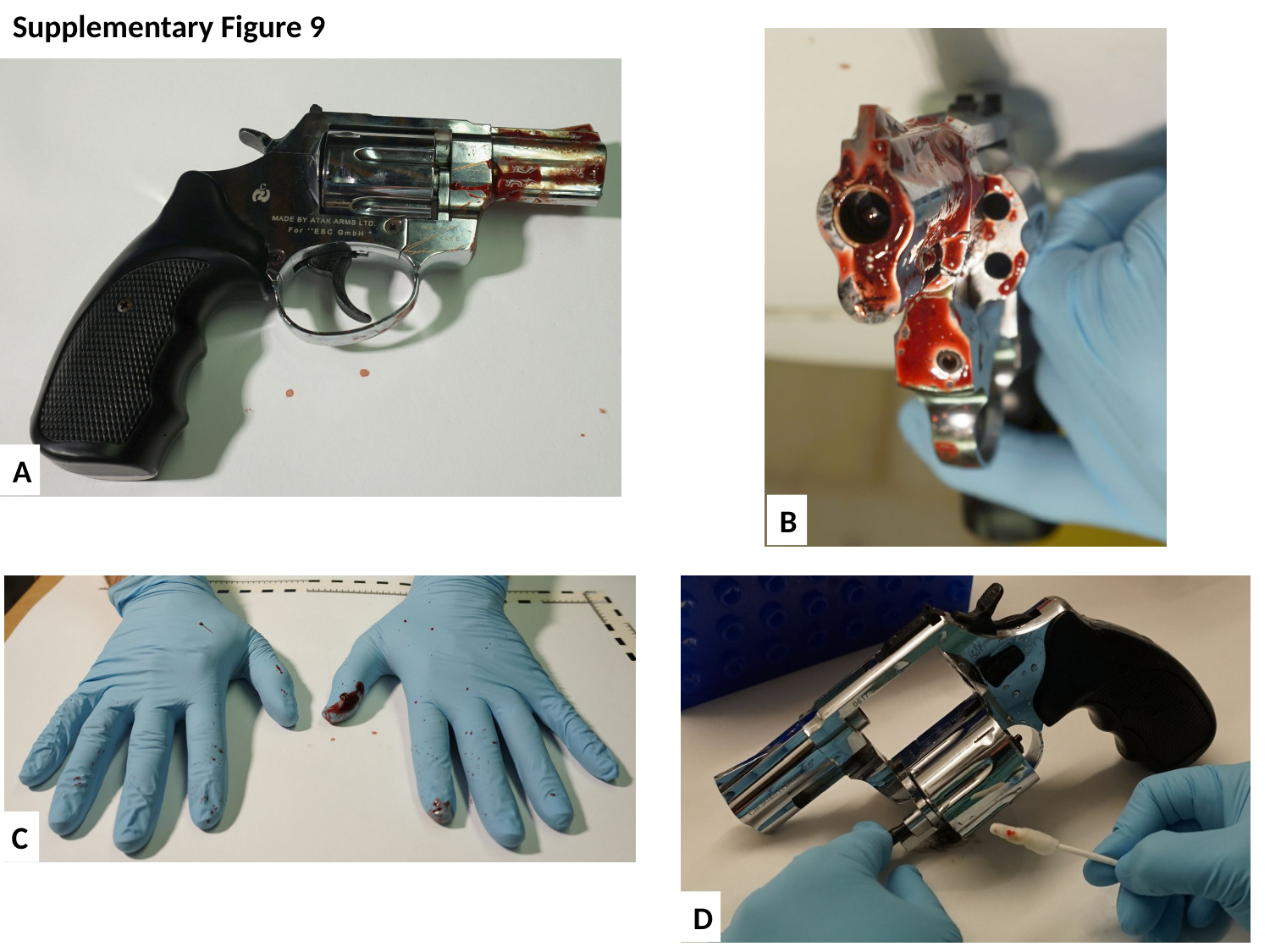

Supplementary Figure 9
A
B
C
D

## Slide 10
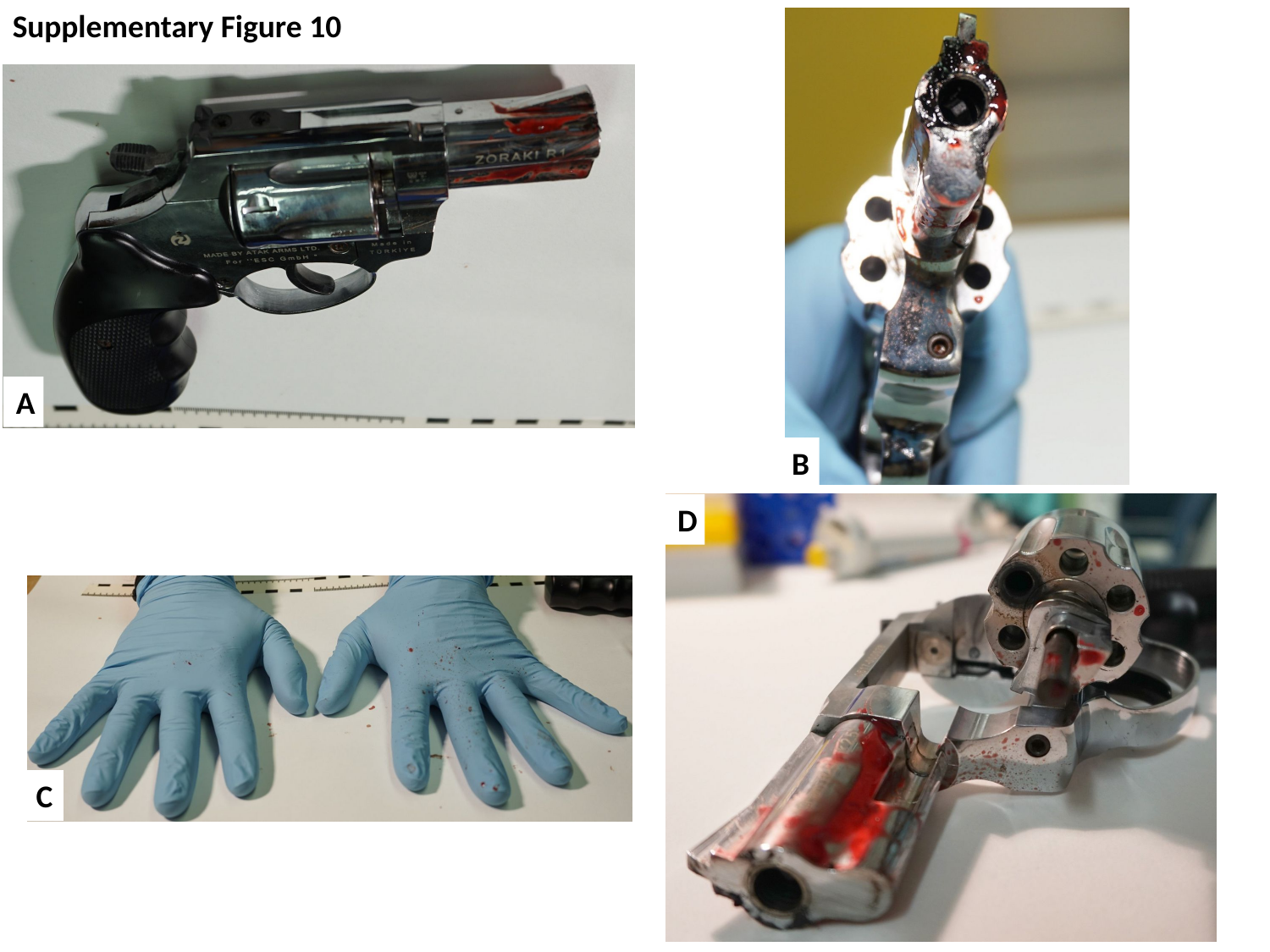

Supplementary Figure 10
A
B
D
C

## Slide 11
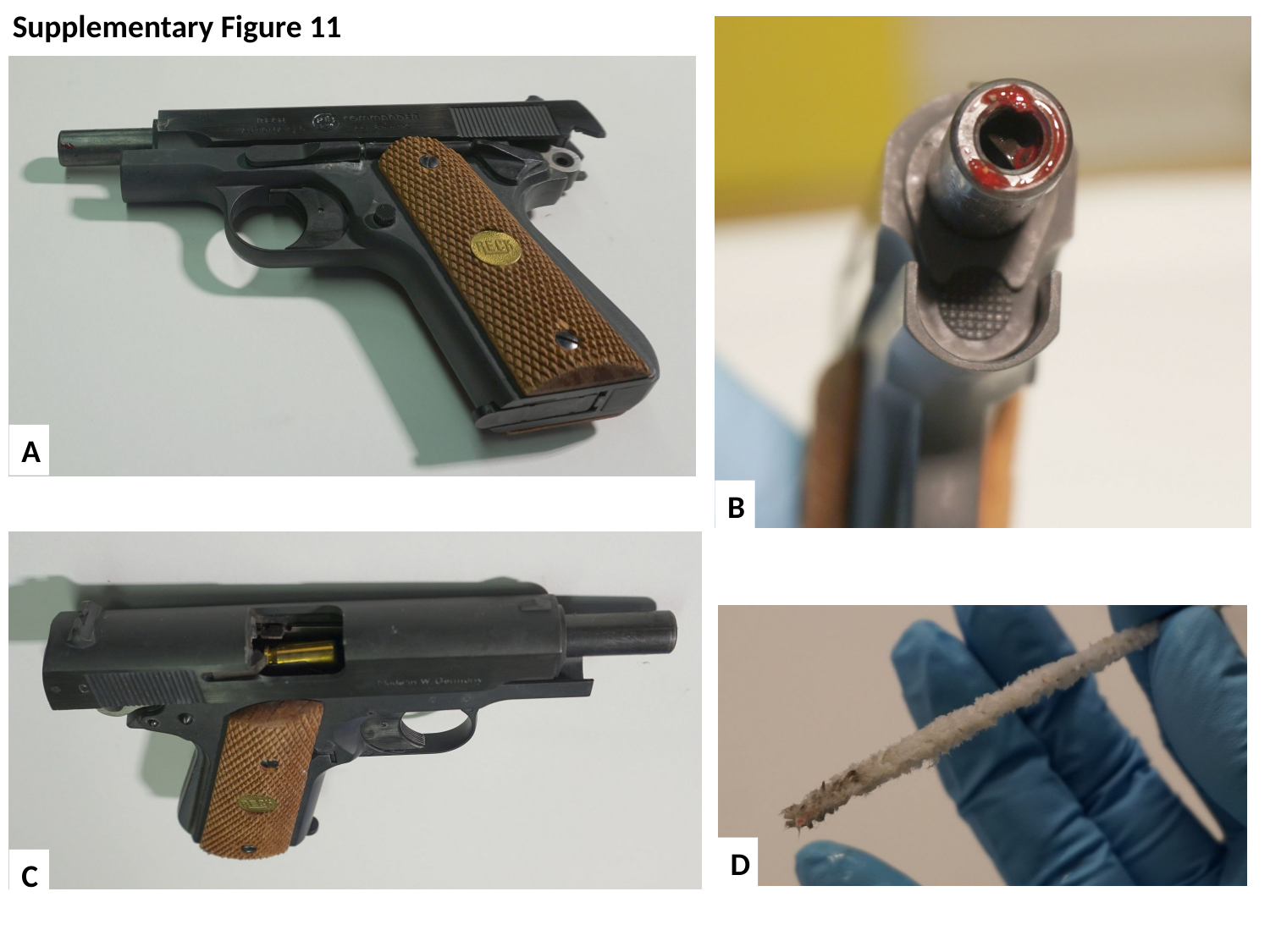

Supplementary Figure 11
A
B
D
C

## Slide 12
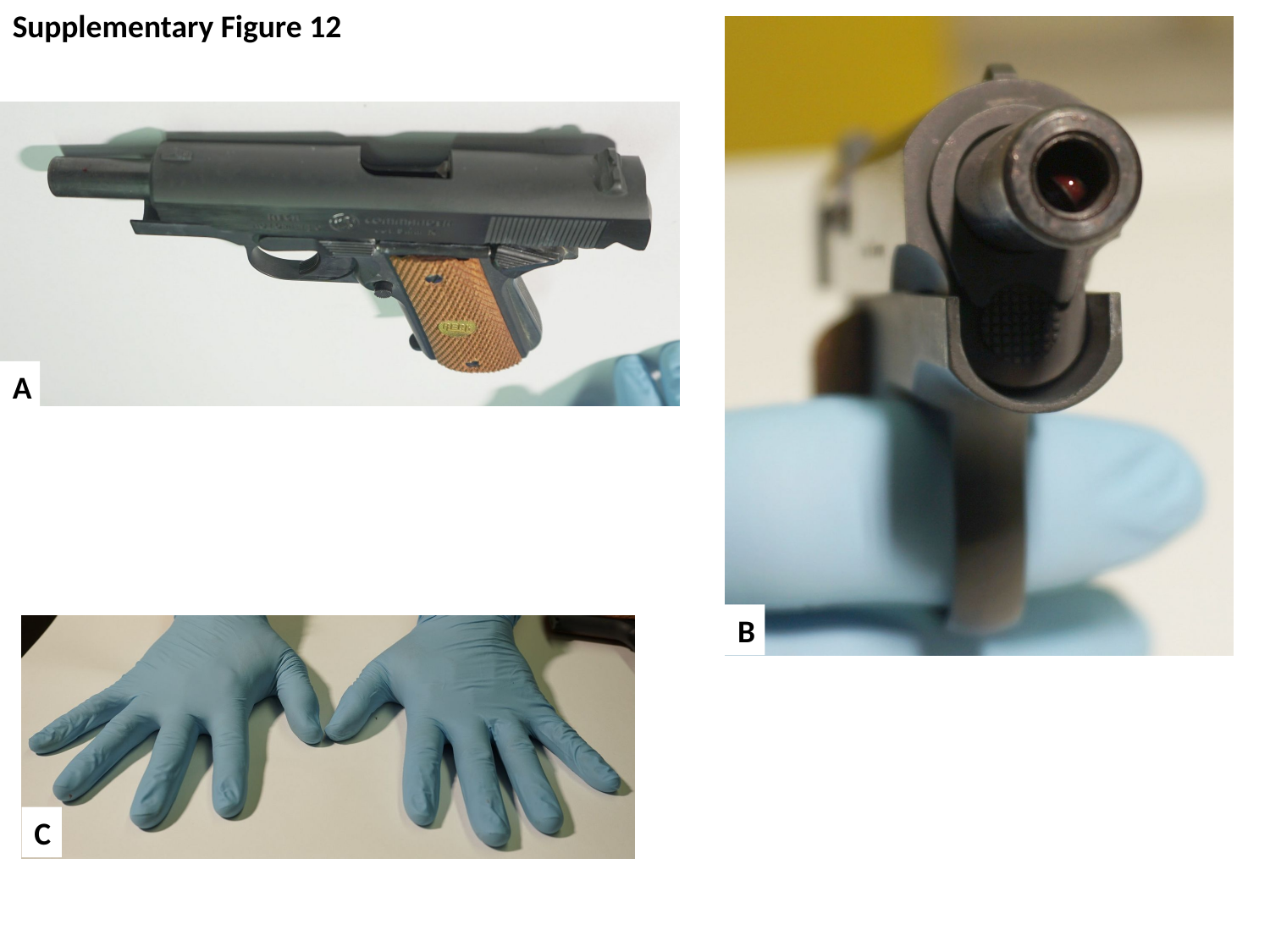

Supplementary Figure 12
A
B
C

## Slide 13
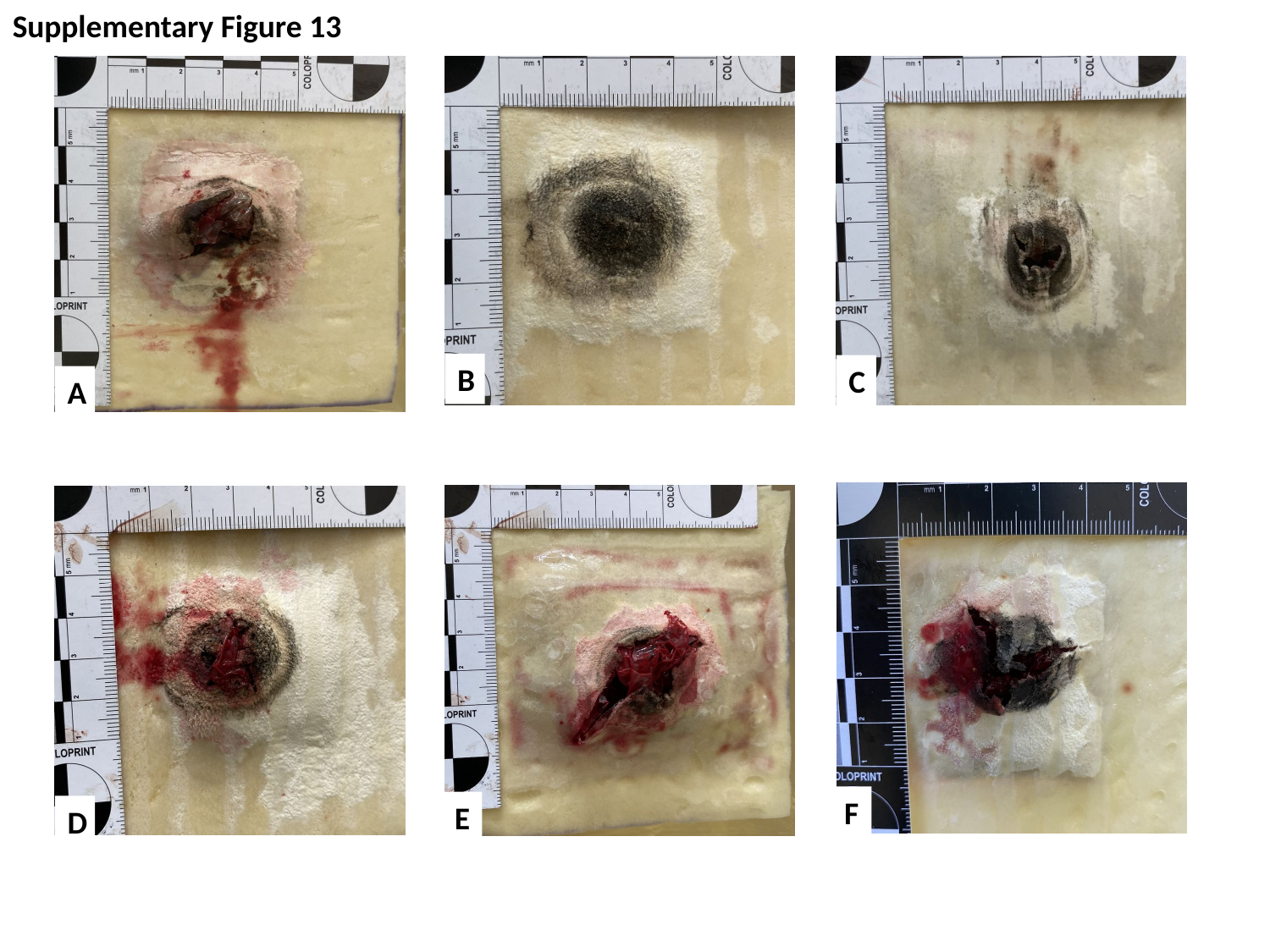

Supplementary Figure 13
B
C
A
F
E
D

## Slide 14
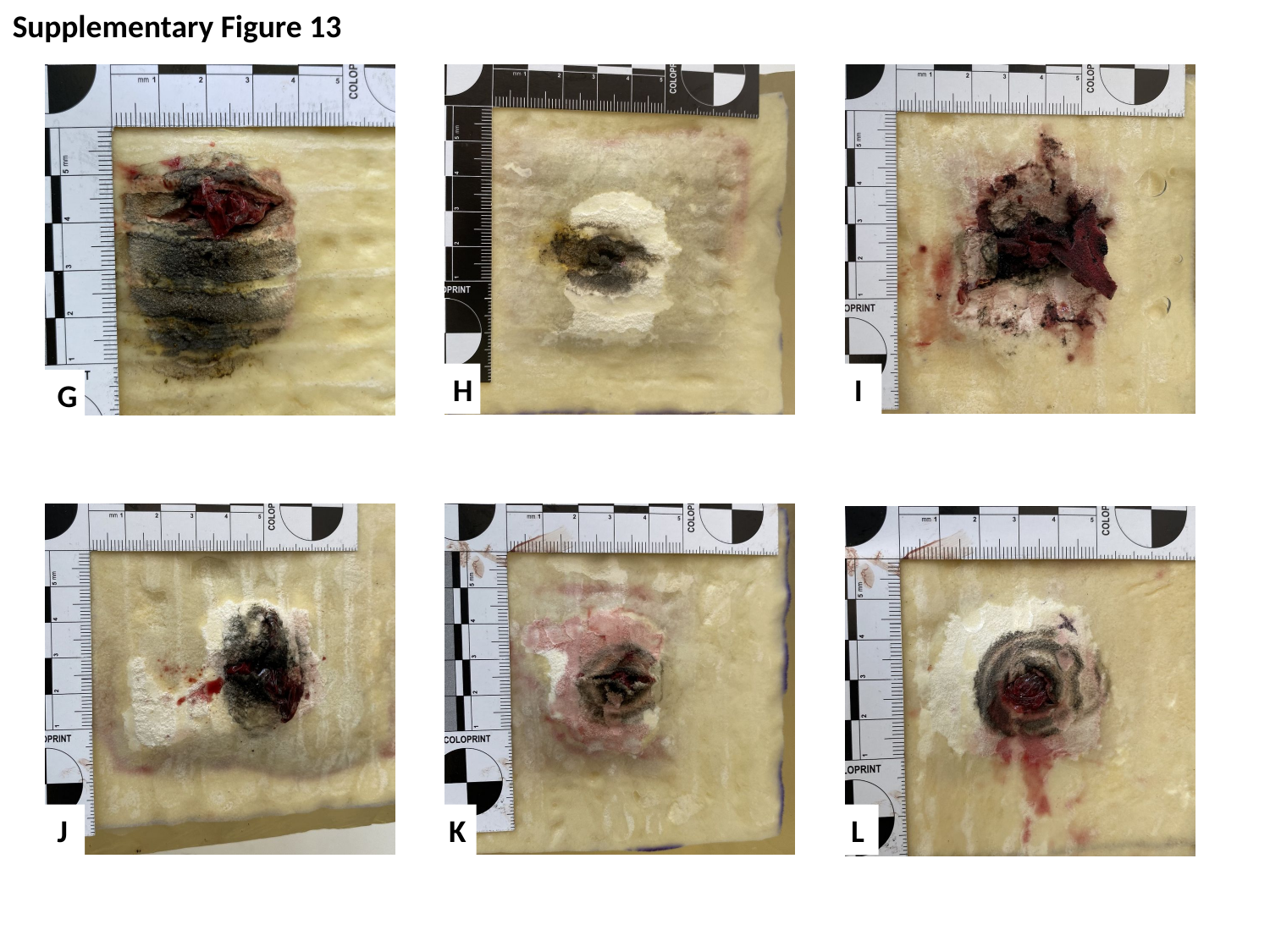

Supplementary Figure 13
H
I
G
J
K
L

## Slide 15
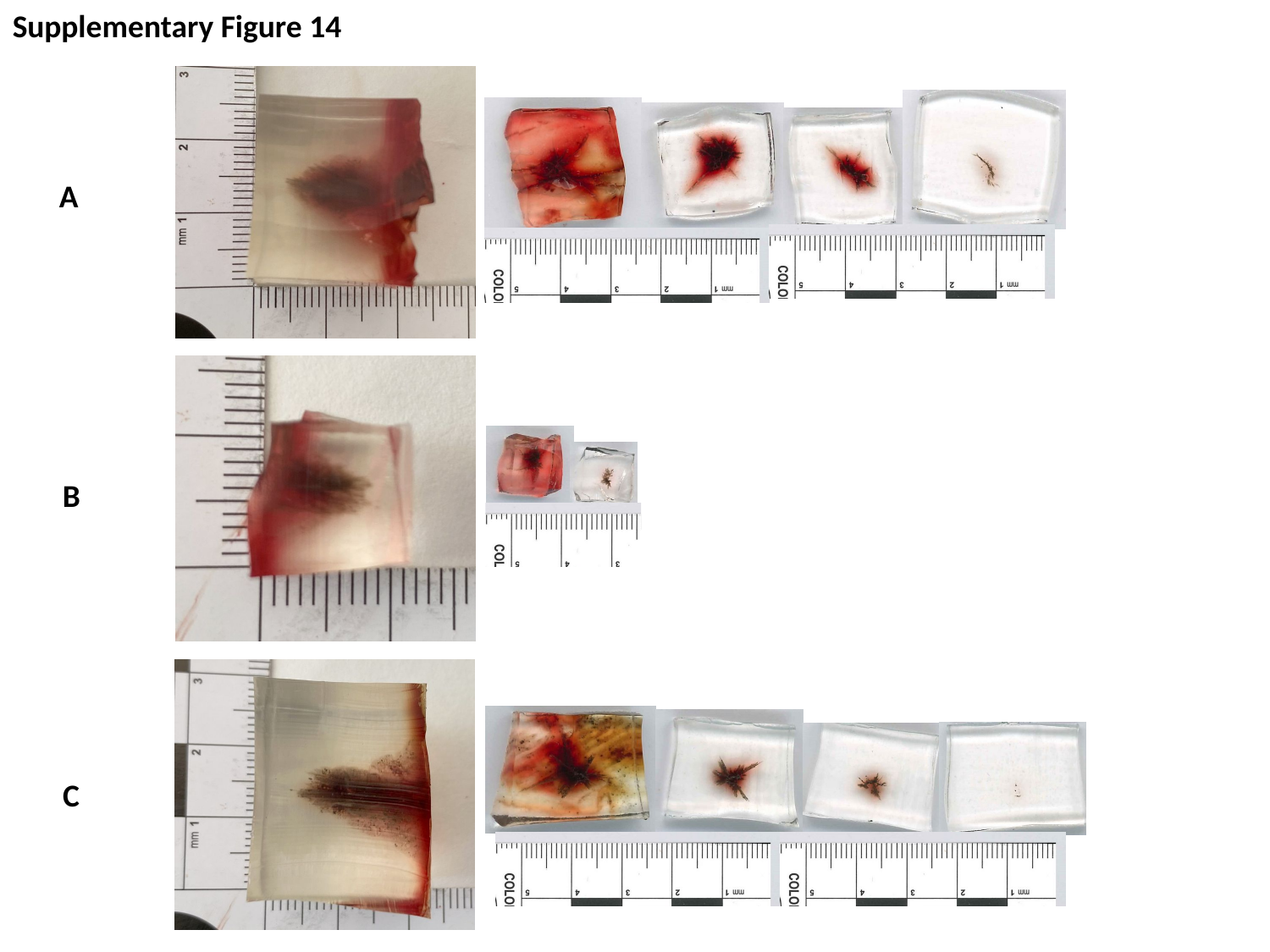

Supplementary Figure 14
A
B
C

## Slide 16
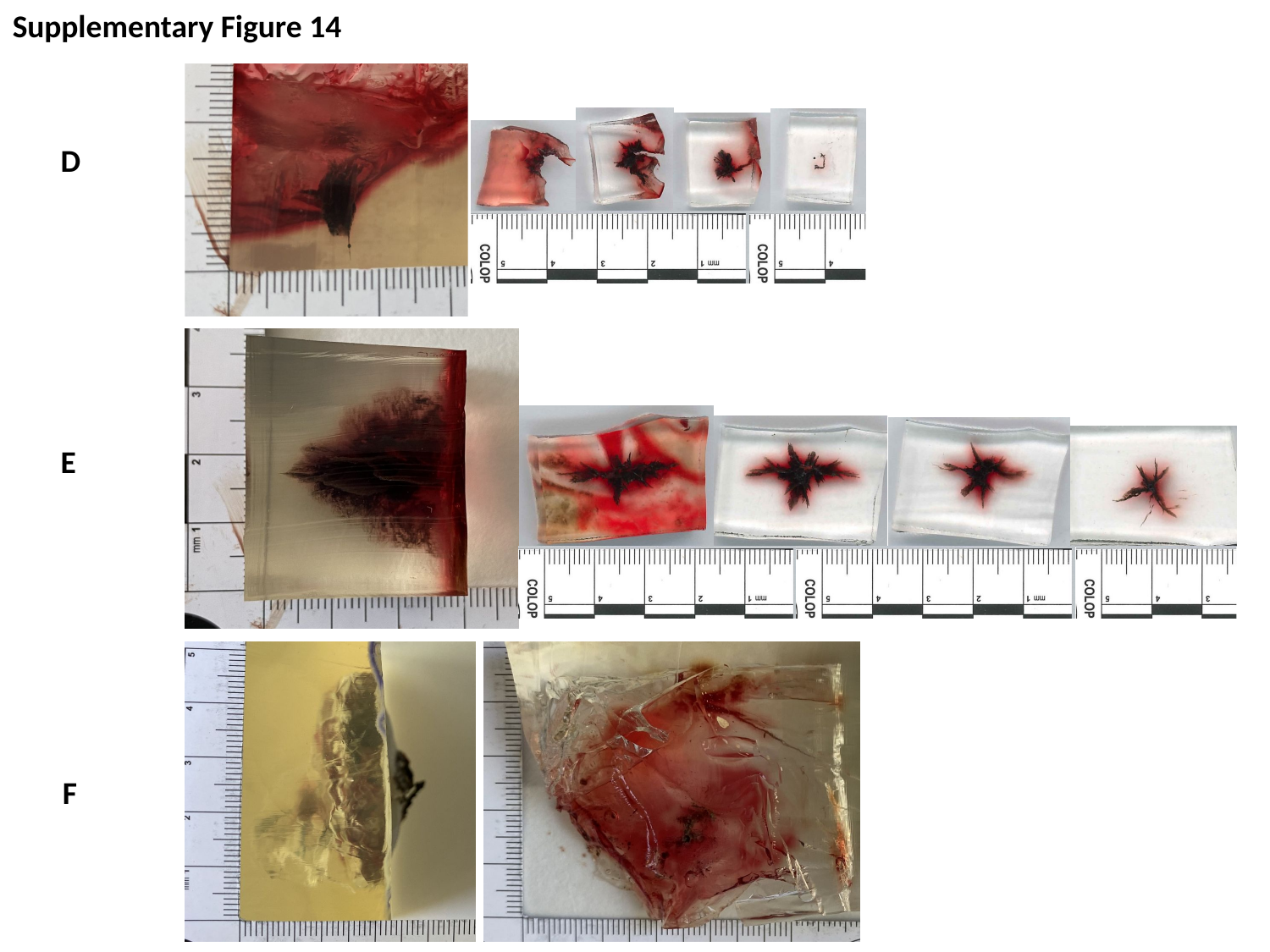

Supplementary Figure 14
D
E
F

## Slide 17
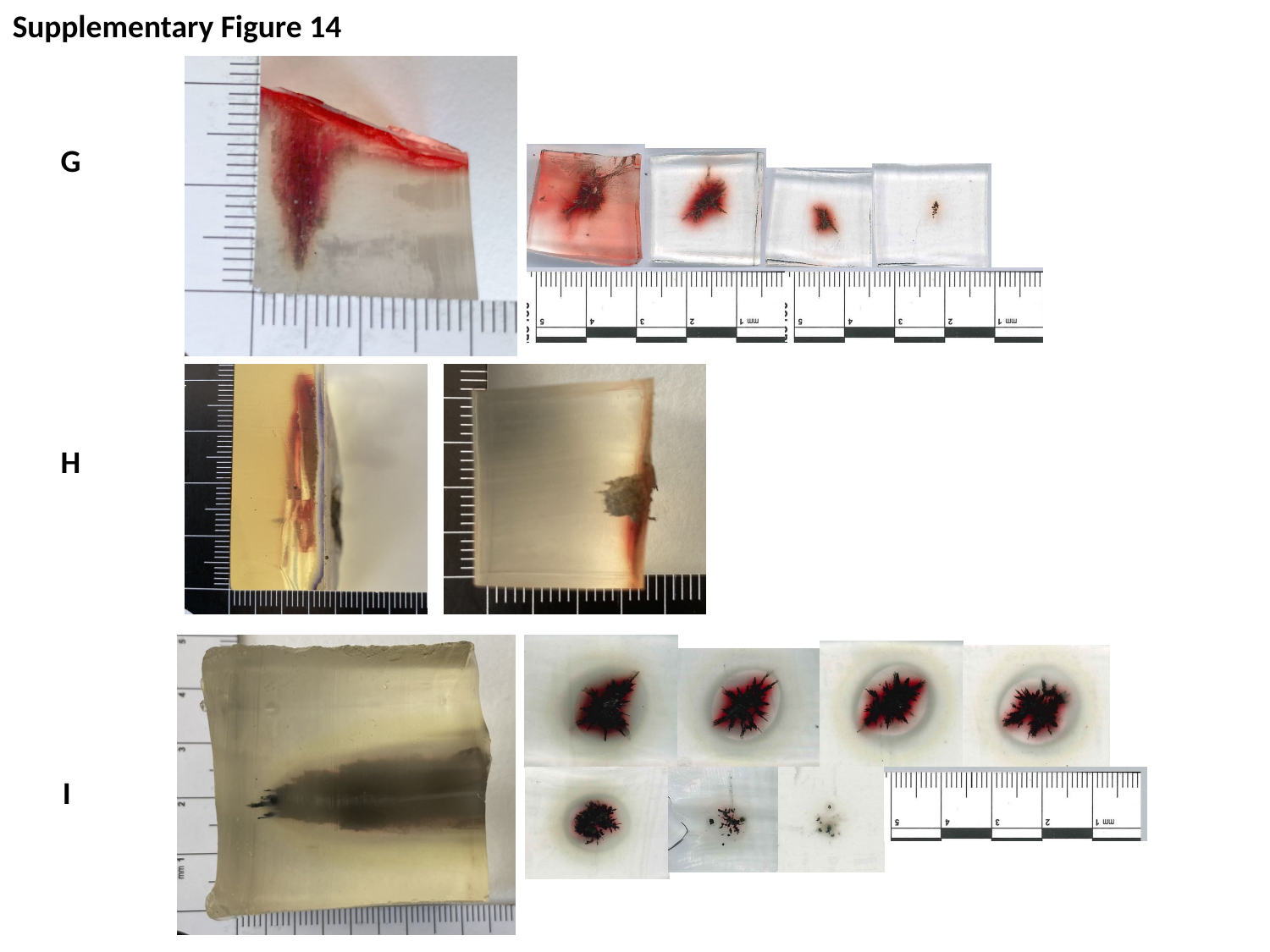

Supplementary Figure 14
G
H
I

## Slide 18
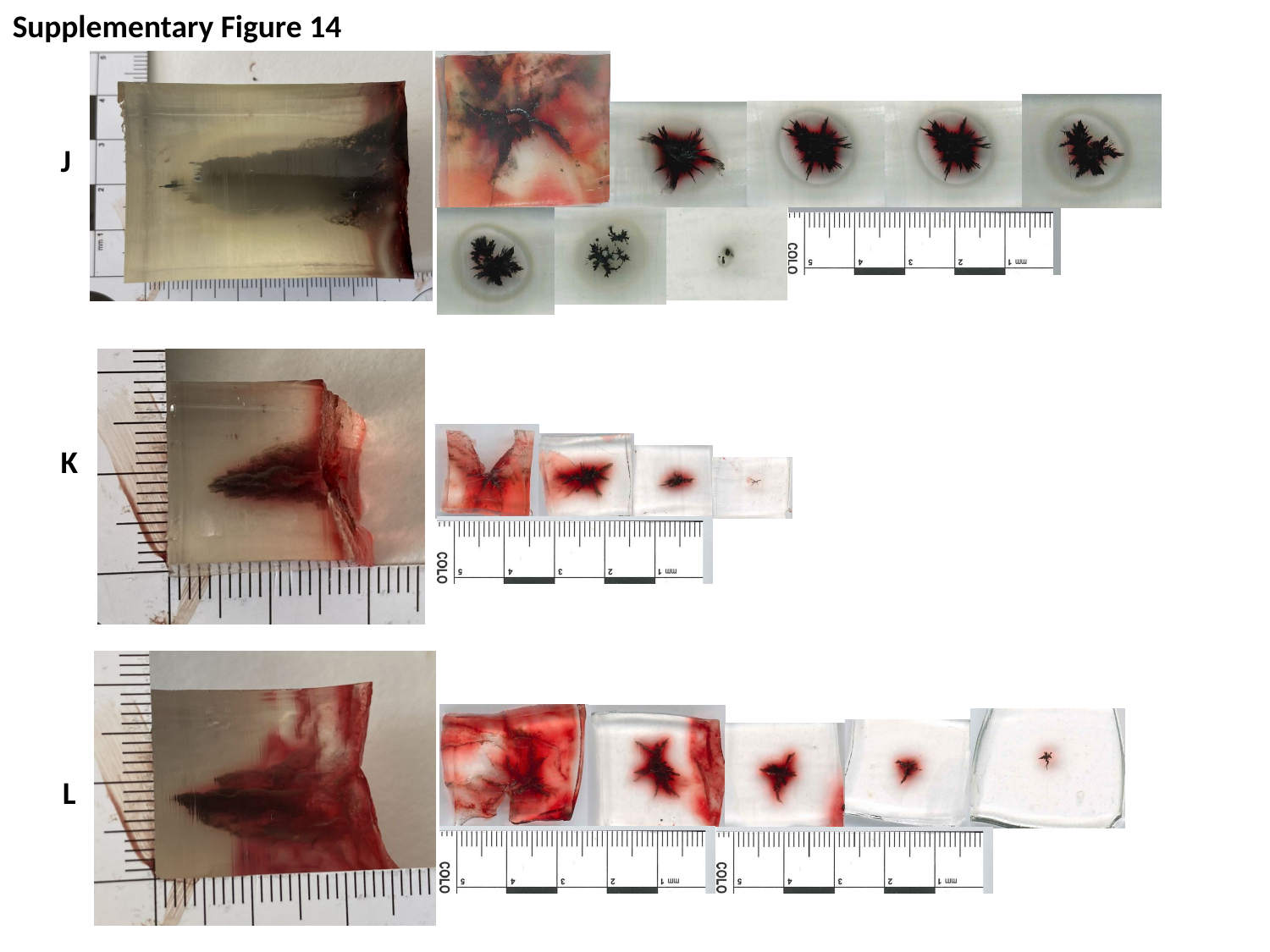

Supplementary Figure 14
J
K
L

## Slide 19
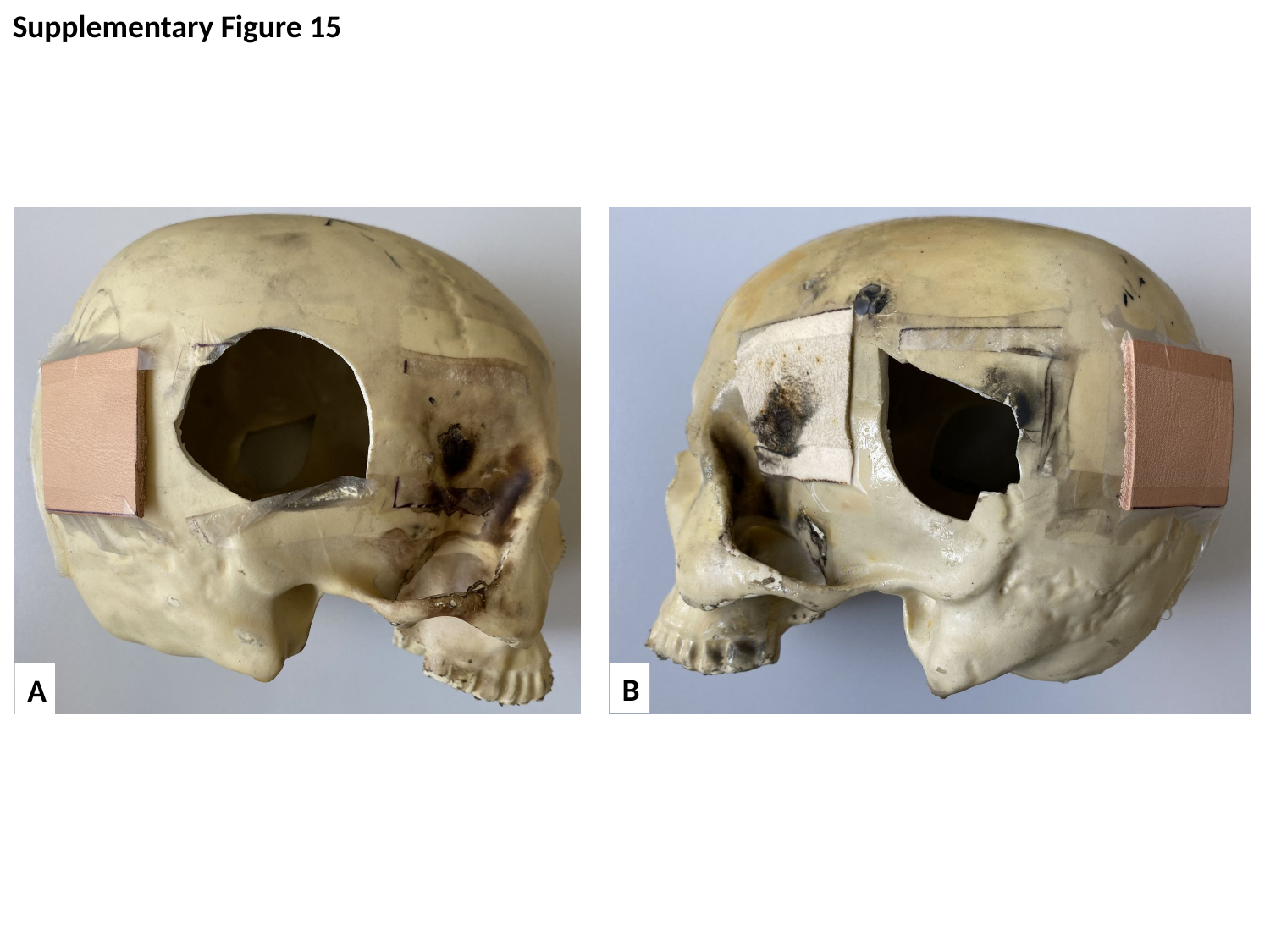

Supplementary Figure 15
B
A

## Slide 20
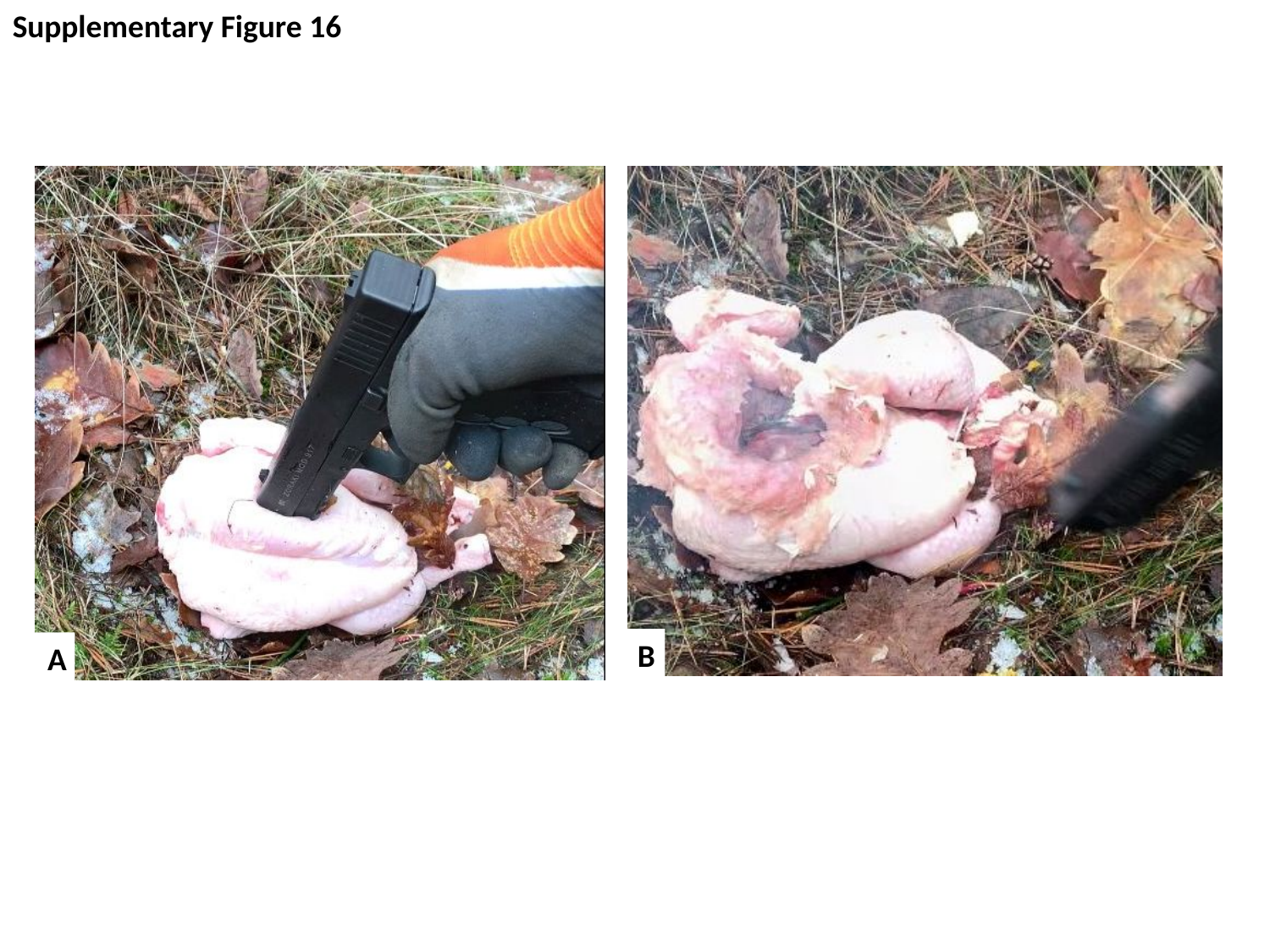

Supplementary Figure 16
B
A

## Slide 21
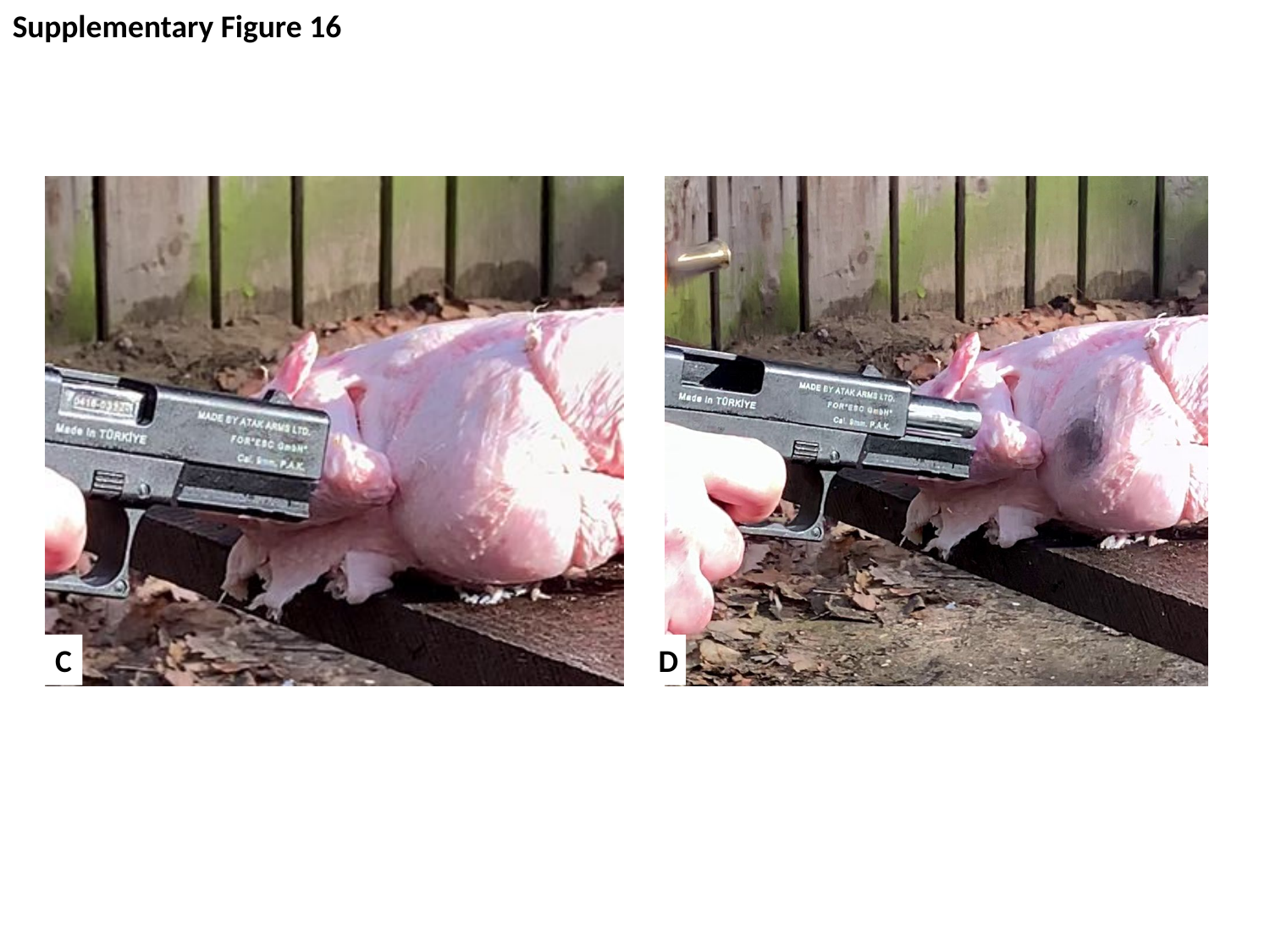

Supplementary Figure 16
C
D

## Slide 22
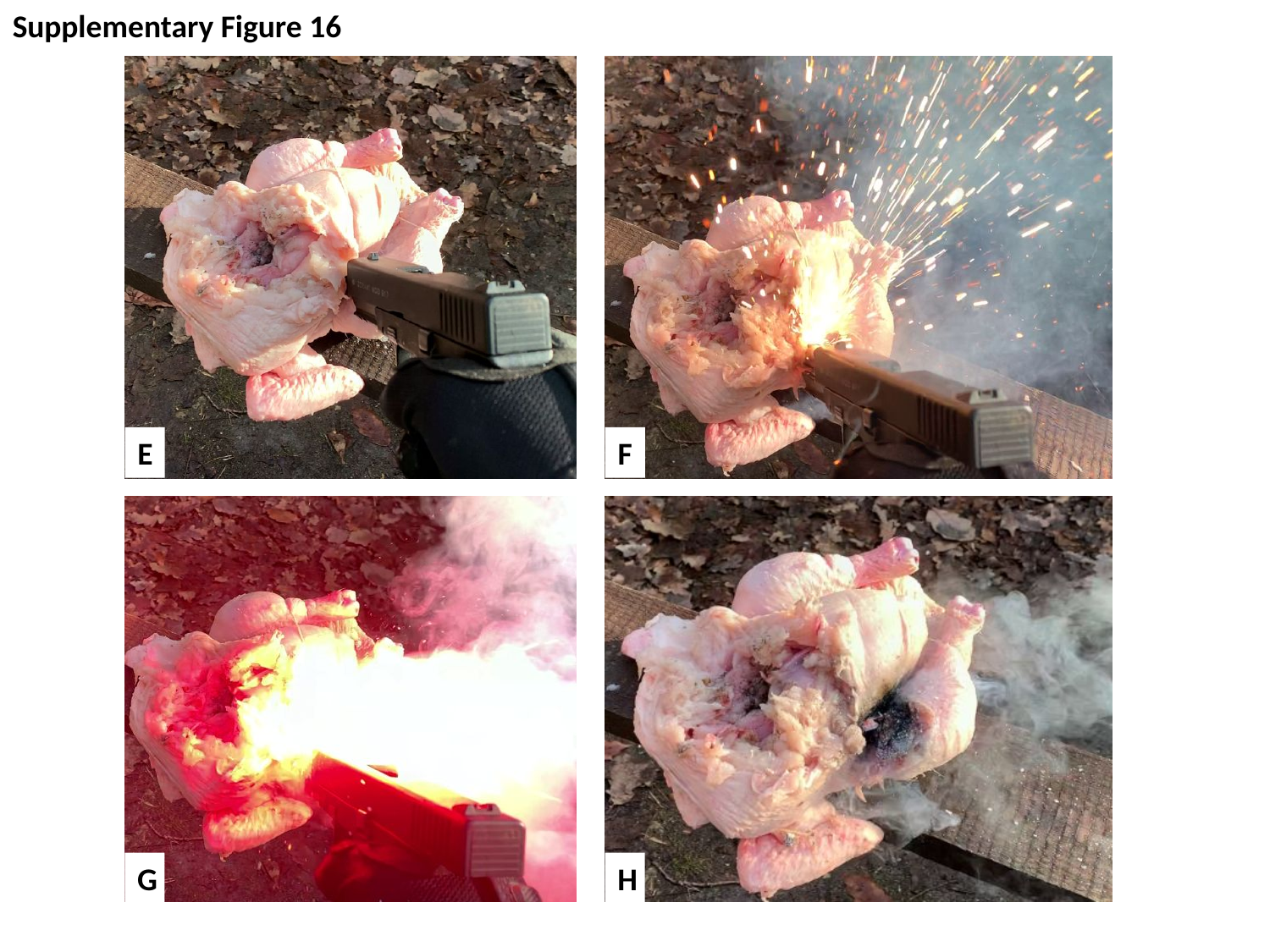

Supplementary Figure 16
E
F
G
H

## Slide 23
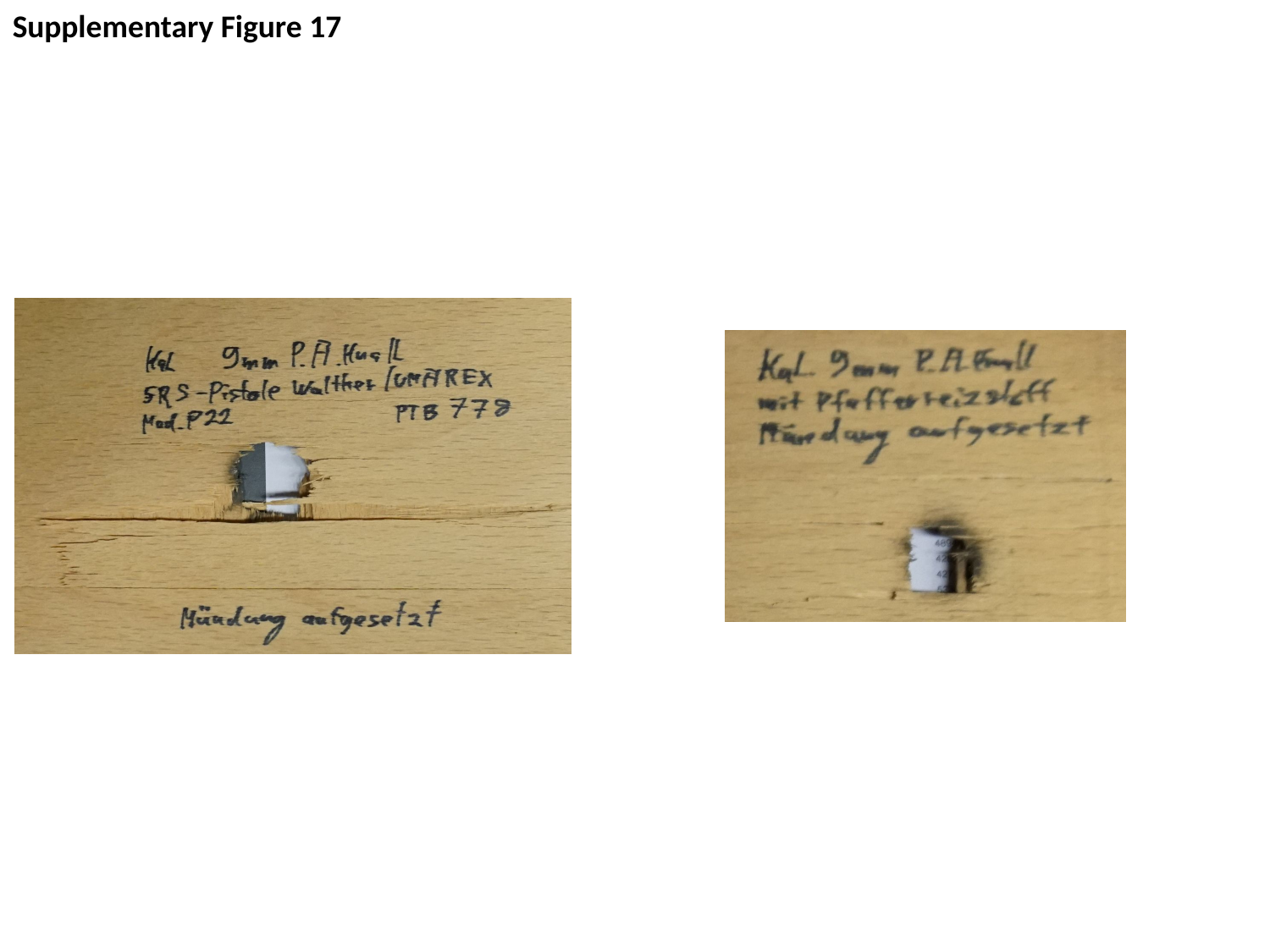

Supplementary Figure 17
