# Supplementary material for: Nothing but hot air?—On the molecular ballistic analysis of backspatter generated by and the hazard potential of blank guns
Source: Int J Legal Med. 2021 Mar 8;135(5):2061–71. doi: 10.1007/s00414-021-02541-y (PMC8354942; doi:10.1007/s00414-021-02541-y)
Supplement: Supplementary file 2 — (DOCX 17 kb) [file 414_2021_2541_MOESM2_ESM.docx]

**Supplementary Material 1 – Negative Controls**

## Evaluation of negative controls

Out of 42 negative controls, 12 (29%) yielded DNA concentrations above 0.4 pg/µL, between 1 and 48.5 pg/µL (Table 1). Of those, 10 originated either from sampling location B or D, i.e. the muzzle area and inside the barrel, where most of the backspatter was detected and effective cleaning had proven to be most difficult due to the barrel blockage. In 7 of 12 cases, STR profiling identified the respective blood donor of the previous shot, indicating that even our thorough and extensive cleaning procedure was insufficient. While this, on the one hand, demonstrates the need to optimize DNA removal when cleaning blank guns, it portends, on the other hand, an opportunity in real cases to retrieve traces from inside the barrel even from guns that had been thoroughly cleaned. In 3 of 12 cases, a mixed profile of two donors with distinct major and minor components were found: in one sample, this was most likely due to contamination, as the minor contributor (all peaks <15% average peak height) had performed the DNA extraction. The other two samples originated from the same shot; there, the minor contributor was the blood donor for the model that had been shot before with the very same weapon. Therefore, minimal traces appeared to have remained inside the weapon even after two rounds of extensive cleaning, although cross contamination during the sample processing cannot be excluded as well. As no contamination was detected at the regular samples collected after the shot, the large amounts of backspatter seem to have ‘quenched’ or masked the minuscule remains of that respective donor. From the last 2 of 12 negative controls, one was a mixed profile with small peaks not clearly attributable to any donor or involved person, and the other represented the STR profile of the shooter, probably due to a contamination while air-drying the weapon after cleaning. There were, however, no STR signals attributable to the shooter found in the following or any subsequent shot from this weapon.

| **Table 1. Summary and evaluation of negative controls with quantifiable DNA amounts** | | | | | | | |
| --- | --- | --- | --- | --- | --- | --- | --- |
| **Weapon** | **Ammunition** | **Model** | **Negative Control** | **Molecular Ballistics Results** | | | **Evaluation** |
| **Manufacturer**  **type - caliber** | **Manufacturer**  **Propellant** | **Setup** | **Sampling Location** | **DNA quantity [ng/µL]** | **Full STR systems from donor** | **Extra alleles** |  |
| Zoraki, R – 9 mm | Walther, NC | 1 | A | 0.0034 | **14/16** | 23 | Extra alleles minor contributor (<15% average peak height), attributable to author doing sample processing. Likely contamination. |
| Zoraki, R – 9 mm | Walther, NC | 1 | B | 0.0275 | **16/16** | - | Unsuccessful cleaning. |
| Zoraki, R – 9 mm | Walther, NC | 1 | D | 0.0062 | **16/16** | 2 | Minor drop-in alleles. Unsuccessful cleaning. |
| Zoraki, R – 9 mm | Walther, NC | 3 | B | 0.0325 | **16/16** | - | Unsuccessful cleaning. |
| Zoraki, R – 9 mm | Geco, NC | 2 | A | 0.0458 | **16/16** | - | Unsuccessful cleaning. |
| Zoraki, R – 9 mm | Geco, NC | 2 | B | 0.0061 | **16/16** | 2 | Minor drop-in alleles. Unsuccessful cleaning. |
| EKOL, P – 9 mm | Skullfire, NC | 2 | B | 0.0010 | 0/16 | **28** | Full STR profile from shooter. Contamination. |
| EKOL, P – 9 mm | Özkursan, NC | 2 | B | 0.0026 | **16/16** | 1 | Minor drop-in alleles. Unsuccessful cleaning. |
| Zoraki, R – 9 mm | Nobel, BP | 1 | A | 0.0287 | **16/16** | 4 | Minor drop-in alleles. Unsuccessful cleaning. |
| Zoraki, R – 9 mm | Nobel, BP | 1 | B | 0.0115 | **16/16** | 15 | Extra alleles minor contributor (<15% average peak height), attributable to donor of block shot before. Likely unsuccessful cleaning. |
| Zoraki, R – 9 mm | Nobel, BP | 1 | D | 0.0010 |  | 26 | Weak profile (63 – 385 RFU) with small peaks, not attributable to one person. Unsuccessful cleaning. |
| Reck, P – 8 mm | Walther, NC | 1 | B | 0.0020 | **9/16** | 6 | All alleles, also extra alleles attributable to donor. Unsuccessful cleaning. |
| For a detailed description of weapons and ammunition, see Table 1. R: Revolver, P: Pistol, NC: Nitrocellulose, BP: Black Powder, Model Setups: 1: single layer chamois leather 2: double layer chamois leather 3: single layer chamois leather + vacuum bag, n.a.: not applicable, Weapon/sampling locations: A: Frame/Slide, B: Muzzle, C: Outer Barrel Surface, D: Inside Barrel. **Bold** in STR systems: major contributor | | | | | | | |
